# A Smart Glove Digital System Promotes Restoration of Upper Limb Motor Function and Enhances Cortical Hemodynamic Changes in Subacute Stroke Patients with Mild to Moderate Weakness: A Randomized Controlled Trial

**DOI:** 10.3390/jcm11247343

**Published:** 2022-12-10

**Authors:** Seyoung Shin, Hwang-Jae Lee, Won Hyuk Chang, Sung Hwa Ko, Yong-Il Shin, Yun-Hee Kim

**Affiliations:** 1 Department of Physical and Rehabilitation Medicine, Center for Prevention and Rehabilitation, Heart Vascular Stroke Institute, Samsung Medical Center, Sungkyunkwan University School of Medicine, Seoul 06351, Republic of Korea; 2 Robot Business Team, Samsung Electronics, Suwon 16677, Republic of Korea; 3 Department of Rehabilitation Medicine, Pusan National University School of Medicine, Pusan National University Yangsan Hospital, Yangsan 50612, Republic of Korea; 4 Research Institute of Convergence for Biomedical Science and Technology, Pusan National University Yangsan Hospital, Yangsan 50612, Republic of Korea; 5 Department of Health Science and Technology, Department of Medical Devices Management and Research, Department of Digital Healthcare, SAIHST, Sungkyunkwan University, Seoul 06355, Republic of Korea

**Keywords:** stroke, upper extremity, motor function, cortical neuroplasticity, virtual reality

## Abstract

This study was a randomized controlled trial to examine the effects of the RAPAEL^®^ Smart Glove digital training system on upper extremity function and cortical hemodynamic changes in subacute stroke patients. Of 48 patients, 20 experimental and 16 controls completed the study. In addition to conventional occupational therapy (OT), the experimental group received game-based digital hand motor training with the RAPAEL^®^ Smart Glove digital system, while the control group received extra OT for 30 min. The Fugl-Meyer assessment (UFMA) and Jebsen-Tayler hand function test (JTT) were assessed before (T0), immediately after (T1), and four weeks after intervention (T2). Cortical hemodynamics (oxyhemoglobin [OxyHb] concentration) were measured by functional near-infrared spectroscopy. The experimental group had significantly better improvements in UFMA (T1-T0 mean [SD]; Experimental 13.50 [7.49]; Control 8.00 [4.44]; *p* = 0.014) and JTT (Experimental 21.10 [20.84]; Control 5.63 [5.06]; *p* = 0.012). The OxyHb concentration change over the ipsilesional primary sensorimotor cortex during the affected wrist movement was greater in the experimental group (T1, Experimental 0.7943 × 10^−4^ μmol/L; Control −0.3269 × 10^−4^ μmol/L; *p* = 0.025). This study demonstrated a beneficial effect of game-based virtual reality training with the RAPAEL^®^ Smart Glove digital system with conventional OT on upper extremity motor function in subacute stroke patients.

## 1. Introduction

Stroke is a leading cause of morbidity and the main cause of sensory-motor impairment worldwide. Several studies have reported that more than 65% of chronic stroke patients have had motor and sensory problems in the hemiparetic upper extremity [1,2]. Hand function is required for many activities of daily living (ADL), such as manipulating objects, eating, and computer and telephone use. Loss of hand function is a serious, common result of a cortical lesion from a cerebrovascular attack [3]. Therefore, recovery of hand function is of primary importance in the neurorehabilitation of stroke survivors. Furthermore, timing must be considered when planning neurorehabilitation focused on neuroplasticity following a stroke [4]. Previous studies have demonstrated that earlier rehabilitation leads to greater neuroplasticity in cortical areas controlling hand function in the lesioned hemispheres [5,6].

Previous evidence suggests that intensive repeated training is likely to be necessary to modify neural organization and promote recovery of hand motor skills in stroke patients [7,8]. Conventional rehabilitation for hand function and neuroplasticity, such as constraint-induced movement therapy [9], high-intensity training, and repetitive task-oriented training [10] often has unsatisfactory results due to insufficient patient motivation. In this regard, game-based virtual reality (VR) training is becoming a promising technology to promote motor recovery by providing high-intensity and repeated task-oriented rehabilitation with three-dimensional game programs involving patient body movement [11,12,13,14]. A 2017 Cochrane Review suggested that addition of VR to conventional care produced a significant difference between intervention and control groups in upper limb function [15]. A recent meta-analysis of 17 studies confirmed the feasibility of VR in early stroke rehabilitation and also suggested that VR intervention showed similar outcomes in upper extremity and ADL function to dose-matched conventional therapy [16].

Additionally, robotic rehabilitation was posited to have a positive effect on attention; reduce the effort needed to enhance motor control, specifically in the hand; boost motivation; boost adherence to treatment; and boost sensorimotor integration [17]. Consequently, robotic rehabilitation may complement standard rehabilitation for restoring hand function [18]. However, despite these advantages, common problems with robotic devices are their high cost, large size, and rigid components. Most devices are designed for hospital use and are too complex for patients to use on their own at home. A sensor-based, soft, smart glove device can allow multiple degrees of freedom and complex motions with soft components [19,20,21]. There are several smart gloves with varying features from exoskeletal gloves to fabric or strip type gloves [22]. Commercial smart gloves have been used in many fields including motion capture, video games, industrial training, and medicine. In this study, we used the RAPAEL^®^ Smart Glove digital system with game-based VR developed by Neofect (Yong-in, Republic of Korea) for task-oriented hand training with interactive motion recognition of user movement. A task-specific, interactive, game-based VR system combined with a soft smart glove can be used for motor recovery in stroke patients [21]. The RAPAEL^®^ Smart Glove digital system with game-based VR system for post stroke patients showed that the smart glove group had greater improvement in upper extremity motor functions than did a conventional occupational therapy group [21,23]. Similar results were demonstrated in a study with cerebral palsy patients [24]. These studies reported consistent results for effectiveness of smart glove systems for upper extremity rehabilitation. However, no study examined cortical biosignals induced by the intervention. The most reliable and repeatable cortical hemodynamic response following motor stimulation consists of increase in oxygenated hemoglobin (OxyHb) coupled with a decrease in deoxygenated hemoglobin (deOxy Hb) [25]. Functional near-infrared spectroscopy (fNIRS) is a useful and non-invasive tool for measuring blood hemodynamic changes by recording the density of cerebral blood oxygenation in real-time [26]. fNIRS is more convenient, less expensive, and more tolerant of patient movement compared to functional magnetic resonance imaging (fMRI) and is more resistant to artifacts than electroencephalography (EEG) [26,27]. Although more evidence is required on the reliability and repeatability of fNIRS data, previous studies suggested fNIRS for localizing cortical activity by measuring hemodynamic changes [25,28].

The aim of this study was to examine the superiority of RAPAEL^®^ Smart Glove digital training compared with conventional occupational therapy (OT) alone on upper extremity function in subacute stroke patients and cortical hemodynamic changes in a small sub-sample of patients.

## 2. Materials and Methods

### 2.1. Participants

A sample size of 40 patients (20 in each of groups) was deemed sufficient to detect a clinically significant difference of 10 points for the Fugl-Meyer assessment (UFMA), assuming a standard deviation of 10.73 points, using a two-sample two-sided *t*-test of mean difference, a power of 80%, and a significance level of 5%. The calculation is based on the assumption that the measurements on UFMA are normally distributed, and the calculations were performed with G*Power software (version 3.1). This study enrolled 48 in-patients with upper limb functional deficits caused by stroke, who presented at Samsung Medical Center of Seoul or Pusan National University Yangsan Hospital of Yangsan-si, Republic of Korea. Participants were eligible for inclusion if they met the following requirements: (1) age between 20 and 85 years, (2) >3 weeks and <3 months after stroke onset, (3) active range of motion (ROM) in the wrist >10 degrees, and (4) unilateral upper limb deficit with a 66 > Fugl-Myer Assessment score >22. Exclusion criteria were (1) history of preexisting neurological or psychiatric disorder, (2) multiple or bilateral stroke lesions, (3) Korean Mini-Mental State Exam (K-MMSE) score <17, (4) aphasia, and (5) pregnancy. Ethics approval was granted by the Ethics Committees of Samsung Medical Center of Seoul and Pusan National University Yangsan Hospital, and written informed consent was obtained from all participants before the study. This study was retrospectively registered at ClinicalTrials.gov (accessed on 1 May 2015, NCT02431390). 

### 2.2. Study Design 

A randomized, controlled, parallel group trial with a single, blinded evaluator design was performed to test the effectiveness of hand motor training with the RAPAEL^®^ Smart Glove digital system and game-based VR in subacute stroke patients. Eligible participants were randomly placed in either the experimental group with hand motor training with the RAPAEL^®^ Smart Glove digital system or in the control group with conventional OT for the same amount of time. All participants were assigned a code number, and a lottery method was used for simple randomization. The clinical research coordinator assigned each patient to one of the two groups after the lottery. The patients were assigned to occupational therapists who conducted the intervention. To ensure blinding of the evaluator, patients were instructed not to share their allocation. The independent examiner who measured the outcomes and the occupational therapists who managed the intervention sessions were experts in their respective fields.

### 2.3. RAPAEL^®^ Smart Glove Digital System

The RAPAEL^®^ Smart Glove digital system was designed to provide repetitive task-oriented training to induce neuroplasticity of the motor system controlling hand function in stroke patients. The system has two types of embedded sensors to collect information on individual motions in real time. From those sensors, the system provides real-time active and passive ROM of the wrist and fingers during movement. Additionally, the system provides information about game-based VR training progress and patient achievements. By applying a ‘Learning Schedule Algorithm’ to game-like exercises, the RAPAEL^®^ Smart Glove can create ADL-related tasks compatible with an individual’s functional level. 

### 2.4. Intervention Protocols

All participants were treated with 20 intervention sessions over 4 weeks, 5 times per week, 1 h per day. The experimental group received game-based VR hand motor training with the RAPAEL^®^ Smart Glove digital system. If participants missed any training during the intervention period, additional sessions were offered at another time during the week or during an optional additional week at the end of the intervention period. For VR treatment, the occupational therapist observed each patient and selected the appropriate content and game level. The participants were required to successfully perform tasks related to a specific intended movement to obtain a high score.

In the training protocol, the average time per session was 1 h, divided into 30 min with the VR training program and 30 min of conventional OT. The intervention structure was customized to participant hand function level. As the sessions progressed, the training intensity gradually increased by changing the VR game level. The control group had 1-h sessions of conventional OT alone without VR hand motor training.

### 2.5. Outcome Measures

We performed the following assessments before intervention (T0), immediately after the intervention (T1), and 4 weeks after the intervention (T2). During outcome measurements, the examiners were blinded to participant group.

#### 2.5.1. Primary Outcome: Motor Function 

An occupational therapist performed upper extremity UFMA for motor impairment of the affected side and the Jebsen-Taylor hand function test (JTT) at T0, T1, and T2. The UFMA consists of 33 items (3-point ordinal scale; range, 0–66), with higher scores indicating less impairment [29]. The JTT assesses hand function according to ADL with a series of 7 timed subtests of writing, simulated page turning, picking up small objects, simulated feeding, stacking checkers, picking up large light objects, and picking up large heavy objects [30]. In the original JTT, a subtest is considered missing if it is not completed within a certain amount of time. To overcome this limitation of the original JTT scoring system, we adopted a modified system presented in a previous study. According to this modification, each subtest is scored from 0 to 15, and the total score is the sum of all subtest scores and ranges from 0 to 105 [31].

#### 2.5.2. Secondary Outcome: Cortical Activation Changes in the Motor Cortical Regions

To investigate cortical hemodynamic changes, we measured OxyHb concentration using the NIRSscout^®^ system (NIRx Medical Technology, Berlin, Germany), which is a multi-modal, compatible, fNIRS platform. This system has optodes of 16 sources and 16 detectors, which cover the primary sensorimotor cortex (SMC), the premotor cortex (PMC), and the supplementary motor area (SMA), using 45 channels of interest (Supplementary Figure S1). The NIRSscout^®^ uses two wavelengths (760 nm and 850 nm) with a sampling rate of 3.91 Hz. The optodes were positioned according to the international 10/20 system, and the channel distance (i.e., distance between the source and detector) was 3.0 cm. Cortical hemodynamic responses were recorded for 700 s at T0 and T1. Baseline OxyHb concentration data were collected during the first 300 s, followed by 400 s of affected wrist and hand movement. This movement consisted of five blocks of 80 s (80 s × 5 times = 400 s), each with 30 s of rest, 10 s of wrist flexion-extension, 30 s of rest, and 10 s of hand grasp task.

Amount of change in OxyHb concentration over the ipsilesional SMC was the secondary outcome. Change in OxyHb concentration was calculated as OxyHb concentration during wrist/hand movement minus OxyHb concentration during rest (ΔOxyHb = OxyHb rest − OxyHb during wrist or hand task). The OxyHb concentration was analyzed by the NIRS-SPM (Near Infrared Spectroscopy-Statistical Parametric Mapping) software package in MATLAB (The Mathworks, MA, USA) [32]. To investigate cortical hemodynamics in the affected side of the brain, the left-brain lesions were flipped from left to right during data preprocessing, so all included lesions were set on the right. We used a modified Beer-Lambert law to calculate OxyHb level following change in cortical concentration [33]. The international 10/20 system was used to position optodes with the cranial vertex (Cz) located beneath the first source. The nasion, left ear, right ear, and inion were identified in each subject. A stand-alone application was used for spatial registration of the 49 functional channels on a Montreal Neurological Institute brain.

Gaussian smoothing with a 2s full width at half maximum (FWHM) was applied to correct noise from the fNIRS system. A wavelet discrete cosine transform (DCT)-based detrending algorithm was used to correct signal distortion due to breathing or movement, and a general linear model (GLM) analysis with a canonical hemodynamic response curve was performed to model the hypothesized OxyHb response under the experimental conditions [32]. To investigate changes in cortical hemodynamics during the affected wrist and hand movements, we selected five regions of interest (ROIs), defined by Brodmann’s area (BA) or anatomical markers: the SMC (BA 1, 2, 3, and 4), the PMC (BA 6), and the SMA (anterior boundary: vertical line to the anterior commissure, posterior boundary: anterior margin of the SMC, medial boundary: midline between the right and left hemispheres, lateral boundary: 15 mm lateral to the midline between the right and the left hemispheres).

### 2.6. Statistical Analysis 

We performed per-protocol (PP) analysis of the data of participants who completed the experiments. All statistical analyses were performed with SPSS version 27.0 (version 27; SPSS, Inc., Chicago, IL, USA), and the significance level was set at 0.05. The Shapiro-Wilk test was used to confirm that all outcome variables were normally distributed. The Chi-square test was used for binary parameters to compare baseline characteristics between groups. For between-group analysis, the independent *t*-test and the Mann-Whitney U test were appropriately used. The paired *t*-test or Wilcoxon signed rank test with Bonferroni’s correction was used for comparing the three time points (T0, T1, and T2). For measuring time x group interaction in UFMA and JTT, repeated measures analysis of variance (RMANOVA) was performed.

For comparing ΔOxyHb, statistical parametric mapping (SPM) t-statistic maps were computed for group analyses and were considered significant at an uncorrected threshold of *p* < 0.05. Median, interquartile range (IQR), and *p* value from the Mann-Whitney U test were provided to depict the change within each group.

## 3. Results

### 3.1. Participants Characteristics 

**Figure 1:**
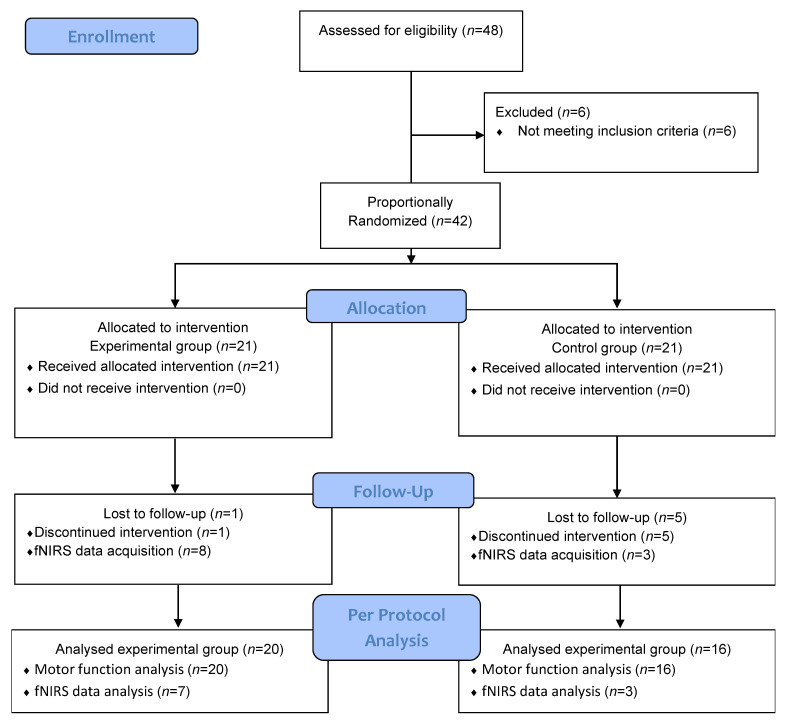
Consort flow diagram. fNIRS: functional near-infrared spectroscopy.

A total of 48 subacute stroke patients were screened for this study from December 2015 to May 2017. Among 48 patients, six did not meet the inclusion criteria. We allocated 42 patients into experimental and control groups, each with 21 patients. During the study, one participant from the experimental group and five from the control group dropped out for reasons of: (1) patient choice (one patient), (2) failure to complete the interventions (three patients), and (3) follow-up loss (two patients). Finally, a total of 36 participants completed the 20-session intervention program and followed up until four weeks after intervention completion. These patients were included for the PP analysis. Figure 1 provides a consort flow diagram of participant recruitment and retention through this study. General characteristics of the 36 participants are shown in Table 1. No significant differences in general characteristics or dependent variables were observed between groups.

### 3.2. Primary Outcome Results

**Figure 2:**
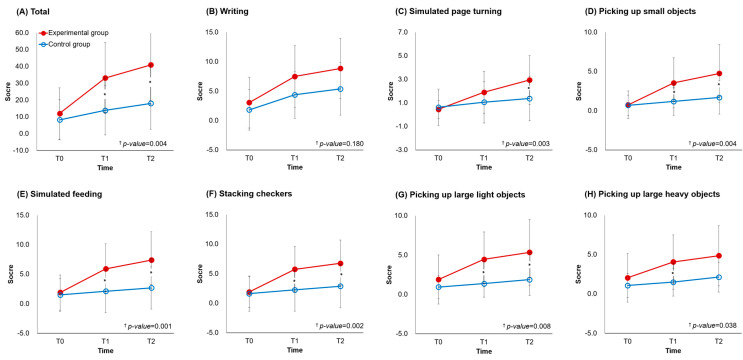
Fugl-Meyer assessment of upper extremity in experimental and control groups. T0, before the intervention; T1, immediately after the intervention; T2, four weeks after the intervention. * *p* < 0.05 between-group comparisons according to independent *t*-test or Mann-Whitney U test for continuous variables as appropriate after Bonferroni’s correction. ^†^ *p*-value according to repeated measure analysis of variance.

The UFMA raw scores before and after intervention are presented in Table 2 (Supplementary Table S1 for median and IQR and Supplementary Table S2 for within-group *p*-value) and Figure 2. The changes in the UFMA scores in the two groups are compared in Table 3. After the intervention, the experimental group showed larger improvement in the UFMA total (mean [SD]; Experimental 13.50 [7.49]; Control 8.00 [4.44]; *p* = 0.014) and subscores in wrist (Experimental 3.35 [2.25]; Control 1.38 [1.36]; *p* = 0.024), hand (Experimental 4.60 [3.68]; Control 2.13 [1.86]; *p* = 0.043), and coordination/speed (Experimental 1.40 [1.14]; Control 0.69 [0.79]; *p* = 0.048) than the control group. In the subsequent four weeks, there were significant differences between the two groups only in UFMA wrist (Experimental 0.20 [1.15]; Control 1.75 [1.44]; *p* = 0.001) subscore. In addition, the UFMA total score had a significant group × time interaction such that the experimental group had greater improvement (*p* < 0.05). Among the UFMA subscores, the wrist and hand items had a significant group × time interaction such that the experimental group showed greater improvement (*p* < 0.05). 

**Figure 3:**
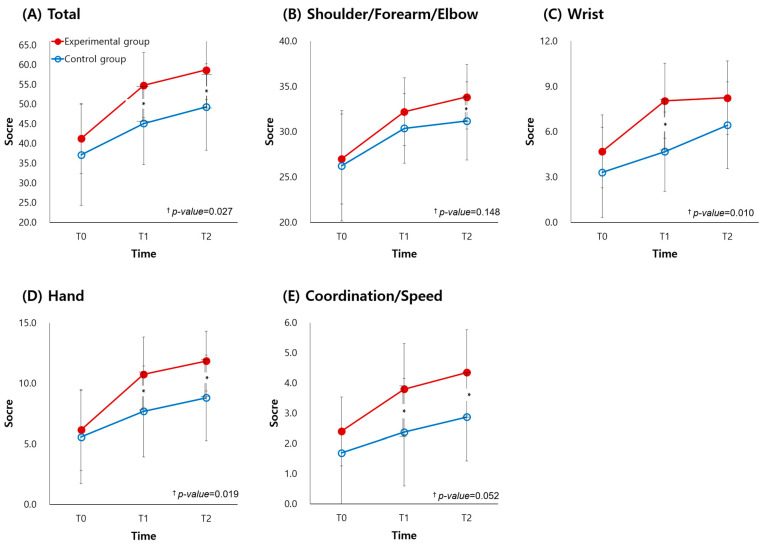
Group analysis of Jebsen-Taylor hand function test in experimental and control groups. T0, before the intervention; T1, immediately after the intervention; T2, four weeks after the intervention. * *p* < 0.05 between-group comparisons according to independent *t*-test or Mann-Whitney U test for continuous variables as appropriate after Bonferroni’s correction. ^†^ *p*-value according to repeated measure analysis of variance.

The JTT raw scores before and after intervention are presented in Table 2 and Figure 3. The changes in JTT scores in the two groups are compared in Table 3. The changes of JTT total (Experimental 21.10 [20.84]; Control 5.63 [5.06]; *p* = 0.012) and subscores in picking up small objects (Experimental 2.80 [3.16]; Control 0.50 [1.10]; *p* = 0.004), simulated feeding (Experimental 4.00 [4.12]; Control 0.63 [1.26]; *p* = 0.003), stacking checkers (Experimental 3.85 [4.04]; Control 0.63 [1.50]; *p* = 0.001), and picking up large light (Experimental 2.55 [2.72]; Control 0..44 [0.51]; *p* = 0.004) and heavy objects (Experimental 2.00 [2.68]; Control 0.44 [0.51]; *p* = 0.041) showed significant differences between groups immediately after the intervention. In the subsequent four weeks, there were significant differences between the two groups only in JTT picking up small objects subscore (Experimental 1.20 [1.15]; Control 0.50 [0.73]; *p* = 0.044). In addition, the JTT total score had a significant group × time interaction such that the experimental group demonstrated greater improvement (*p* < 0.05). More interestingly, for each individual JTT component (simulated page turning, picking up small objects, simulated feeding, stacking checkers, picking up large light objects, and picking up large heavy objects) except for writing, the experimental group showed significantly greater improvement than the control group (*p* < 0.05), according to RMANOVA.

### 3.3. Secondary Outcome Results

fNIRS data were gathered from 11 consenting participants (eight experimental and three control). One patient from the experimental group was excluded from the final analysis because of background noise. In total, seven participants from the experimental group and three from the control group were included for analysis. Statistical parametric mapping revealed t-statistic maps for ΔOxyHb concentration during wrist and hand movement. 

**Figure 4:**
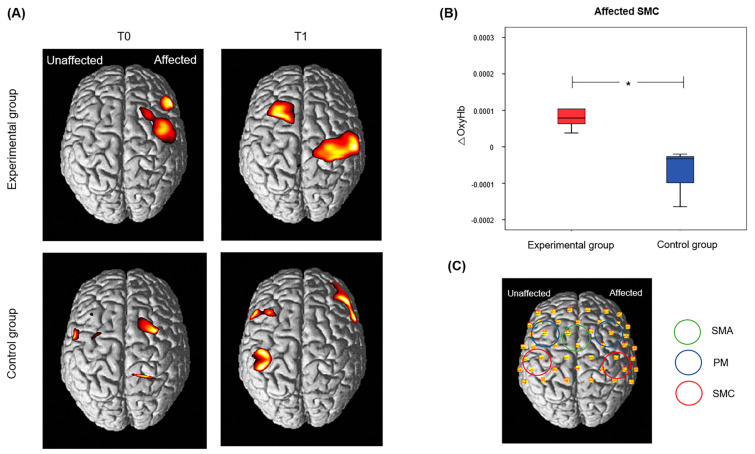
The results of group analysis of oxygenated hemoglobin in experimental and control groups during affected wrist movement. (**A**) Group-average activation map of OxyHb. (**B**) Changes in oxygenated hemoglobin concentration (ΔOxyHb) in the affected primary sensorimotor cortex at T1. (**C**) fNIRS channel montage and affected cortical areas. T0: before the intervention; T1: immediately after the intervention; OxyHb: oxygenated hemoglobin; SMC: primary sensorimotor cortex; PMC: premotor cortex; SMA: supplementary motor area. * *p* < 0.01 for between-group comparison (Mann-Whitney U test).

Immediately after the intervention (T1), ΔOxyHb concentration in the affected SMC was greater in the experimental group than the control group during affected wrist movement (median values; Experimental group, 0.7943 × 10^−4^ μmol/L; Control group, −0.3269 × 10^−4^ μmol/L; *p* = 0.025) (Figure 4). On the other hand, ΔOxyHb values showed no significant differences in any cortical area during finger movement or in the unaffected SMC, bilateral PMC, or SMA during wrist movement (Supplementary Table S3).

## 4. Discussion

The current study was conducted to examine the effects of neurorehabilitation with the RAPAEL^®^ Smart Glove digital system on upper extremity motor function in subacute stroke patients. The findings from this study suggest that convergent VR training with the RAPAEL^®^ Smart Glove digital system combined with conventional OT has some key benefits in terms of upper extremity neurorehabilitation compared with conventional OT only. The game contents used in this study were closely related to ADLs and might provide a positive effect not only on motor function, but also on ADL performance. More importantly, recovery in upper extremity motor function and ADLs was maintained for four weeks after the intervention. 

It is well known that activation of the contralesional hemisphere could be an obstacle for motor recovery of the affected hand in the early or subacute stage of stroke [34,35]. On the other hand, activation of the ipsilesional hemisphere is a main therapeutic strategy for motor recovery. Successful rehabilitation and neurostimulation have been shown to increase ipsilesional cortical activation [36]. In other words, suppressing cortical activity of the unaffected areas and facilitating cortical activity of the affected areas are key for motor recovery after stroke. In this study, fNIRS data of sub-samples of the study population showed that game-based VR training with the RAPAEL^®^ Smart Glove digital system can possibly increase hemodynamic changes of the affected SMC. Although this is weak evidence because of the limited participants, the results suggest that game-based VR training can induce positive cortical hemodynamic changes in subacute stroke patients. This finding must be verified in a larger study population. 

In general, cortical changes result from changes in behavioral patterns, an important finding in neurorehabilitation [37]. Intensive rehabilitation training in subacute stroke patients induced changes in cortical sensorimotor maps and maximized improvement in motor function [38]. A previous functional magnetic resonance imaging (fMRI) study reported that activation of the affected SMC was significantly increased in a virtual environment [39]. VR stimulation also induced differences in connectivity of brain cortices in healthy participants [40]. A recent review article introduced several studies using VR for upper limb rehabilitation in stroke patients, since it is a promising tool for encouraging active engagement of participants to lead to good outcomes [41]. However, one study showed only low to moderate results of VR techniques in subacute stroke patients [42]. Our results indicate not only positive effects of the RAPAEL^®^ Smart Glove digital system on upper limb function, but also possible evidence for underlying cortical hemodynamic changes detected by fNIRS.

In a previous study, robot-assisted therapy showed a comparable effect to conventional therapy, while VR showed no superiority to conventional therapy when provided alone [42]. However, both new techniques showed significantly better functional improvement when they were added to conventional therapy [18,42,43]. The RAPAEL^®^ Smart Glove digital system collects haptic data from a human body and provides real-time ROM information. The intervention protocol of this study was a combined therapy of a smart glove with VR and conventional occupational therapy which provides evidence of the usefulness of such combination therapy protocol.

In rehabilitation, patient motivation and convenience are important in achieving a positive clinical outcome [44]. User-friendly equipment will foster patient use. A smart glove has the advantages of convenience, light weight, availability for haptic stimulation, and remote control. A smart glove can be used in an unsupervised environment such as the home to allow telerehabilitation [45].The RAPAEL^®^ Smart Glove digital system is wearable, lightweight, and flexible for hand movement [19]. None of the participants in this study who completed 20 training sessions with the RAPAEL^®^ Smart Glove digital system experienced adverse events. This result indicates that this system does not pose a risk or cause discomfort to patients, while increasing their motivation. Therefore, the RAPAEL^®^ Smart Glove digital system has a potential to be applied safely both in and out of the clinic.

This study had some limitations. First, the number of participants used in analysis was relatively small. Although we allocated enough participants after sample size calculation, PP analysis was performed for a smaller number of patients due to drop-out (Figure 1). Second, fNIRS analyses were performed in a small number of participants, contributing to a low statistical power of cortical hemodynamic results. The numbers in the experimental and control groups showed discrepancies and did not follow a normal distribution. Therefore, caution is needed when generalizing these results to all subacute stroke patients. Nevertheless, our study has the advantage of real-time biosignal measurements of cortical hemodynamic changes, which provides a clue for further investigation of brain activity related through VR devices. Future research should confirm the neuroplastic effect of the RAPAEL^®^ Smart Glove digital system in a sufficient number of participants. The usability of the VR-based smart glove for home-based rehabilitation also needs to be examined to expand its usefulness for upper extremity dysfunction in stroke patients. 

## 5. Conclusions

This study demonstrated a beneficial effect of combined game-based VR training with the RAPAEL^®^ Smart Glove digital system with conventional OT on upper extremity motor function in subacute stroke patients. In addition, the VR-based smart glove combined with conventional rehabilitation showed a possibility of increasing cortical hemodynamic changes in the affected SMC of these patients.

## Figures and Tables

**Table 1:** Baseline characteristics of the study population.

Characteristic	Experimental Group (*n* = 20)	Control Group (*n* = 16)	*p*-Value
Sex (male: female)	10:10	7:9	0.709 ^b^
Age (years)	57.00 (12.78)	63.69 (8.58)	0.070 ^a^
Stroke onset duration (days)	24.70 (16.26)	34.00 (25.49)	0.336 ^c^
Stroke type			
Ischemic: Hemorrhagic	11:09	13:03	0.097 ^b^
Side of stroke			
Right: left	13:07	9:07	0.593 ^b^
Upper extremity function			
UFMA, total	41.30 (8.90)	37.13 (12.84)	0.258 ^a^
JTT, total	12.00 (15.29)	8.25 (11.93)	0.290 ^c^
Spasticity of upper extremity			
MAS 0: MAS 1	18:02	15:01	0.585 ^b^
K-MMSE	24.95 (3.97)	25.31 (3.57)	0.778 ^a^

Continuous values are expressed as mean (standard deviation). UFMA, Fugl-Meyer assessment of upper extremity; JTT, Jebsen-Taylor hand function test; MAS, Modified Ashworth Scale; K-MMSE, Korean version of the Mini-Mental State Exam. ^a^ Independent *t*-test; ^b^ Chi-square test; ^c^ Mann-Whitney test.

**Table 2:** Assessment scores for the affected upper extremity in the experimental and control groups.

Variables	Experimental Group (*n* = 20)			Control Group (*n* = 16)			Time × Group Interaction
	T0	T1	T2	T0	T1	T2	*p*-Value
UFMA, total	41.30 (8.90)	54.80 (8.27) ^a^	58.70 (7.53) ^b,c^	37.13 (12.84)	45.13(10.44) ^a^	49.32 (10.98) ^b,c^	0.027 *
UFMA, subscore							
Shoulder/Elbow/Forearm	27.00 (4.95)	32.20 (3.73) ^a^	33.85 (3.54) ^b,c^	26.25 (6.10)	30.38 (3.85) ^a^	31.19 (4.31) ^b^	0.148
Wrist	4.70 (2.41)	8.05 (2.48) ^a^	8.25 (2.45) ^b^	3.31 (2.98)	4.69 (2.63) ^a^	6.44 (2.87) ^b,c^	0.010 *
Hand	6.15 (3.34)	10.75 (3.09) ^a^	11.85 (2.48) ^b,c^	5.56 (3.85)	7.69(3.75) ^a^	8.81 (3.54) ^b,c^	0.019 *
Coordination/Speed	2.40 (1.14)	3.80 (1.51) ^a^	4.35 (1.42) ^b,c^	1.69 (1.85)	2.38 (1.78) ^a^	2.88 (1.45) ^b^	0.052
JTT, total	12.00 (15.29)	33.10 (21.12) ^a^	40.90 (23.52) ^b,c^	8.25 (11.93) ^a^	13.88 (14.62)	18.00 (15.41) ^b,c^	0.004 **
JTT, subscore							
Writing	3.05 (4.32)	7.50 (5.24) ^a^	8.85 (5.13) ^b,c^	1.81 (3.43)	4..38 (4.02) ^a^	5.38 (4.47) ^b,c^	0.180
Simulated page turning	0.45 (0.76)	1.90 (1.78) ^a^	2.95 (2.06) ^b,c^	0.63 (1.54)	1.01 (1.77) ^a^	1.38 (1.89) ^b^	0.003 **
Picking up small objects	0.75 (1.77)	3.55 (3.19) ^a^	4.75 (3.70) ^b,c^	0.69 (1.30)	1.19 (1.80)	1.69 (2.12) ^b^	0.004 **
Simulated feeding	1.90 (2.97)	5.90 (4.25) ^a^	7.40 (4.80) ^b,c^	1.50 (2.81)	2.13 (3.61)	2.69 (3.57) ^b^	0.001 **
Stacking checkers	1.90 (2.59)	5.75 (3.88) ^a^	6.75 (3.93) ^b^	1.63 (2.96)	2.25 (3.59)	2.88 (3.67) ^b^	0.002 **
Picking up large light objects	1.90 (3.11)	4.45 (3.49) ^a^	5.35 (4.17) ^b,c^	0.94 (1.48)	1.38 (1.71) ^a^	1.88 (2.00) ^b^	0.008 **
Picking up large heavy objects	2.05 (3.10)	4.05 (3.44) ^a^	4.85 (3.83) ^b^	1.06 (1.53)	1.50 (1.79) ^a^	2.13 (1.89) ^b^	0.038 *

All values are presented as mean (SD). T0: before the intervention; T1: immediately after the intervention; T2: four weeks after the intervention; UFMA: Fugl-Meyer assessment of upper extremity; JTT: Jebsen-Taylor hand function test. * *p* < 0.05, ** *p* < 0.01 for time x group interaction by repeated measurement analysis of variance (RMANOVA). ^a^ *p* < 0.05 for T1-T0, ^b^ *p* < 0.05 for T2-T0, ^c^ *p* < 0.05 for T2-T1 within-group comparisons by paired *t*-test or Wilcoxon signed rank test as appropriate after Bonferroni’s correction. The uncorrected and corrected *p*-values are described in Supplementary Table S2.

**Table 3:** Changes in assessment scores for the affected upper extremity in the experimental and control groups.

Variables	T1-T0			T2-T1		
	Experimental	Control	*p*-Value	Experimental	Control	*p*-Value
UFMA, total	13.50 (7.49)	8.00 (4.44)	0.014 *	3.90 (2.55)	4.19 (3.71)	0.488
UFMA, subscore						
Shoulder/Elbow/Forearm	5.20 (3.22)	4.13 (2.75)	0.297	1.65 (1.79)	0.81 (1.91)	0.362
Wrist	3.35 (2.25)	1.38 (1.36)	0.024 *	0.20 (1.15)	1.75 (1.44)	0.001 **
Hand	4.60 (3.68)	2.13 (1.86)	0.043 *	1.10 (1.33)	1.13 (1.09)	0.716
Coordination/Speed	1.40 (1.14)	0.69 (0.79)	0.048 *	0.55 (0.69)	0.50 (0.89)	0.587
JTT, total	21.10 (20.84)	5.63 (5.06)	0.012 *	7.80 (6.21)	4.13 (3.84)	0.073
JTT, subscore						
Writing	4.45 (5.06)	2.56 (3.24)	0.374	1.35 (1.53)	1.00 (1.10)	0.519
Simulated page turning	1.45 (1.85)	0.44 (0.51)	0.083	1.05 (1.36)	0.31 (0.60)	0.075
Picking up small objects	2.80 (3.16)	0.50 (1.10)	0.004 **	1.20 (1.15)	0.50 (0.73)	0.044 *
Simulated feeding	4.00 (4.12)	0.63 (1.26)	0.003 **	1.50 (1.82)	0.56 (1.21)	0.067
Stacking checkers	3.85 (4.04)	0.63 (1.50)	0.001 **	1.00 (1.81)	0.63 (0.96)	0.647
Picking up large light objects	2.55 (2.72)	0.44 (0.51)	0.004 **	0.90 (1.33)	0.50 (0.73)	0.451
Picking up large heavy objects	2.00 (2.68)	0.44 (0.51)	0.041 *	0.80 (1.32)	0.63 (1.15)	0.713

All values are presented as mean (SD). T0: before the intervention; T1: immediately after the intervention; T2: four weeks after the intervention; UFMA: Fugl-Meyer assessment of upper extremity; JTT: Jebsen-Taylor hand function test. * *p* < 0.05, ** *p* < 0.01 for between-group comparisons by independent *t*-test or Mann-Whitney U test as appropriate.

## Data Availability

The data that support the findings of this study are available from the corresponding author on reasonable request.

## Supplementary Materials

The following supporting information can be downloaded at: https://www.mdpi.com/article/10.3390/jcm11247343/s1. Table S1. Median scores of the Fugl-Meyer assessments for affected upper extremity in the experimental and control groups. Table S2. Uncorrected and corrected *p*-values of within-group analysis. Table S3. Changes in oxygenated hemoglobin concentration in brain cortices. Figure S1. Arrangement of channels in functional near-infrared analysis.
