# Supplementary material for: A Smart Glove Digital System Promotes Restoration of Upper Limb Motor Function and Enhances Cortical Hemodynamic Changes in Subacute Stroke Patients with Mild to Moderate Weakness: A Randomized Controlled Trial
Source: J Clin Med. 2022 Dec 10;11(24):7343. doi: 10.3390/jcm11247343 (PMC9782087; doi:10.3390/jcm11247343)
Supplement: Supplementary file 1 [file jcm-11-07343-s001.zip › jcm-2028432-supplementary.pdf]

Supplementary Figure S1. Arrangement of channels in the functional near-infrared spectroscopy (fNIRS) NIRSscout® system (NIRx Medical Technology, Berlin, Germany).

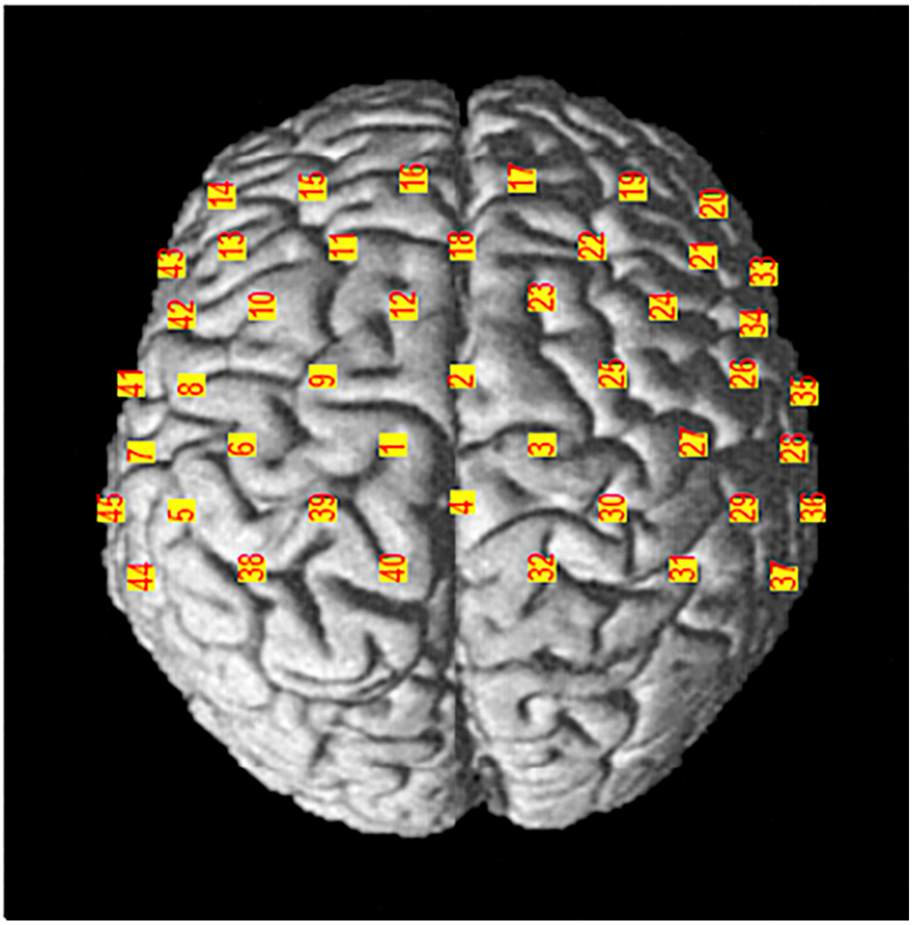

Supplementary Table S1. Median scores of the Fugl-Meyer assessments for affected upper extremity in the experimental and control groups.

Variables	Experimental group (*n* = 20)			Control group (*n* = 16)		
	T0	T1	T2	T0	T1	T2
UFMA, total	42.00 (5.00)	55.00 (8.00)	60.50 (7.00)	35.50 (17.00)	44.50 (8.00)	50.00 (9.00)
UFMA, subscore						
Shoulder/Elbow/Forearm	27.00 (7.75)	33.50 (4.75)	35.00 (2.00)	27.00 (9.25)	30.50 (5.00)	32.00 (5.00)
Wrist	5.00 (2.00)	9.00 (3.00)	9.00 (2.75)	2.50 (2.75)	5.00 (2.75)	7.00 (3.50)
Hand	7.00 (4.25)	10.50 (6.00)	13.00 (4.00)	4.50 (3.50)	7.50 (2.75)	9.00 (2.75)
Coordination/Speed	2.00 (1.00)	4.00 (2.75)	4.50 (3.00)	1.00 (3.00)	2.00 (2.75)	2.50 (1.75)

All values are presented as median (interquartile range). T0: before the intervention; T1: immediately after the intervention; T2: 4 weeks after the intervention; UFMA: Fugl-Meyer assessment of upper extremity.

Supplementary Table S2. Uncorrected and corrected *p*-values of within-group analysis.

Variables	Uncorrected *p-values*						Corrected *p-values*					
	Experimental (*n* = 20)			Control (*n* = 16)			Experimental (*n* = 20)			Control (*n* = 16)		
	T0-T1	T0-T2	T1-T2	T0-T1	T0-T2	T1-T2	T0-T1	T0-T2	T1-T2	T0-T1	T0-T2	T1-T2
UFMA, total	<0.001	<0.001	<0.001	<0.001	<0.001	<0.001	<0.001	<0.001	0.001	<0.001	<0.001	<0.001
UFMA, subscore												
Shoulder/Elbow/Forearm	<0.001	0.001	0.0005	0.001	0.001	0.112	<0.001	0.002	0.0015	0.002	0.002	0.337
Wrist	<0.001	<0.001	0.389	0.001	<0.001	<0.001	0.001	<0.001	1.000	0.003	<0.001	<0.001
Hand	<0.001	<0.001	0.003	0.004	0.001	0.003	0.001	0.001	0.009	0.011	0.004	0.010
Coordination/Speed	<0.001	<0.001	0.002	0.009	0.003	0.035	<0.001	<0.001	0.006	0.028	0.009	0.104
JTT, total	<0.001	<0.001	0.005	0.001	0.001	0.001	0.001	<0.001	0.014	0.004	0.003	0.004
JTT, subscore												
Writing	<0.001	<0.001	<0.001	0.003	0.001	0.007	0.001	<0.001	0.001	0.009	0.003	0.021
Simulated page turning	0.002	<0.001	0.005	0.008	0.002	0.059	0.006	0.001	0.014	0.024	0.007	0.176
Picking up small objects	0.001	<0.001	0.001	0.066	0.010	0.023	0.003	0.001	0.002	0.197	0.029	0.069)
Simulated feeding	0.001	<0.001	0.002	0.020	0.004	0.071	0.002	0.001	0.006	0.059	0.012	0.212
Stacking checkers	<0.001	<0.001	0.023	0.116	0.003	0.020	0.001	<0.001	0.069	0.349	0.009	0.060
Picking up large light objects	0.001	0.001	0.011	0.008	0.002	0.023	0.002	0.002	0.032	0.024	0.007	0.069
Picking up large heavy objects	0.004	0.002	0.017	0.008	0.004	0.034	0.011	0.005	0.051	0.024	0.012	0.101

All values are presented as uncorrected *p-value* or corrected *p-value*. T0: before the intervention; T1: immediately after the intervention; T2: 4 weeks after the intervention; UFMA: Fugl-Meyer assessment of upper extremity; JTT: Jebsen-Taylor hand function test.

Supplementary Table S3. Changes in oxygenated hemoglobin concentration in brain cortices

Brain Lesion \ △OxyHb (μmol/L) \ Group	Experimental Group (*n* = 7)		Control Group (*n* = 3)†		Between-group *p-value*
	T0	T1	T0	T1	
**Wrist movement-Baseline**					
SMC					
Affected	1.3834×10^{-4} (0.5896×10^{-4})	0.7943×10^{-4} (0.4124×10^{-4})	-0.3700×10^{-4}	-0.3269×10^{-4}	0.025*
Unaffected	1.4553×10^{-4} (1.4047×10^{-4})	1.4194×10^{-4} (2.2862×10^{-4})	0.1164×10^{-4}	1.3104×10^{-4}	0.881
PMC					
Affected	1.2188×10^{-4} (0.5198×10^{-4})	0.6617×10^{-4} (0.6105×10^{-4})	-0.2130×10^{-4}	-0.2500×10^{-4}	0.655
Unaffected	0.6519×10^{-4} (0.9242×10^{-4})	0.4494×10^{-4} (0.4616×10^{-4})	0.5818×10^{-4}	0.0425×10^{-4}	0.606
SMA	0.4534×10^{-4} (1.1716×10^{-4})	0.7369×10^{-4} (1.7467×10^{-4})	0.6102×10^{-4}	-0.4189×10^{-4}	0.439
**Finger movement-Baseline**					
SMC					
Affected	$0. 7820 \times 10^{-4}$ ($0. 9164 \times 10^{-4}$)	$0. 9307 \times 10^{-4}$ (0.3966×10^{-4})	0.0475×10^{-4}	0.8661×10^{-4}	0.881
Unaffected	$0. 4493 \times 10^{-4}$ (1.1226×10^{-4})	0.5127×10^{-4} (3.2622×10^{-4})	0.1023×10^{-4}	0.0741×10^{-4}	0.655
PMC					
Affected	2.0866×10^{-4} (2.5215×10^{-4})	1.2101×10^{-4} (0.5846×10^{-4})	-0.6377×10^{-4}	0.7058×10^{-4}	0.180
Unaffected	$0. 4942 \times 10^{-4}$ (1.1178×10^{-4})	0.0774×10^{-4} (0.5085×10^{-4})	0.5057×10^{-4}	0.9533×10^{-4}	0.439
SMA	0.6580×10^{-4} (1.3520×10^{-4})	-0.1704×10^{-4} (1.4498×10^{-4})	-0.3257×10^{-4}	-0.0839×10^{-4}	0.796

All values are presented as median (interquartile range) unless mentioned otherwise; △OxyHb: Changes in oxygenated hemoglobin concentration; T0: before the intervention; T1: immediately after the intervention; SMC: sensorimotor cortex; PMC: premotor cortex; SMA: supplementary motor area.

†Only median values were presented.

*$p < 0.05$ according to Mann-Whitney U test.
